# The benefit of surgery during systematic therapy for gastrointestinal stromal tumor liver metastasis: a SEER-based retrospective study

**DOI:** 10.1093/gastro/goae095

**Published:** 2024-11-25

**Authors:** Bozhi Hu, Yingjiang Ye, Zhidong Gao

**Affiliations:** Department of Gastrointestinal Surgery, Peking University People’s Hospital, Beijing, P. R. China; Department of Gastrointestinal Surgery, Peking University People’s Hospital, Beijing, P. R. China; Department of Gastrointestinal Surgery, Peking University People’s Hospital, Beijing, P. R. China

**Keywords:** GIST liver metastasis, SEER, surgery

## Abstract

**Background:**

The liver is the most common site of gastrointestinal stromal tumor (GIST) metastasis. Most patients who develop metastases gradually develop multiline drug resistance during long-term systematic treatment. We aimed to evaluate the benefit of surgery during the systematic treatment of GIST liver metastases.

**Methods:**

Data on GISTs with liver metastasis were retrieved from the Surveillance, Epidemiology, and End Results database. This study included 607 patients, of whom 380 patients were treated with chemotherapy alone (Chemo group) and 227 patients underwent surgery in addition to chemotherapy (Chemo&Surg group). The primary outcomes were cancer-specific survival (CSS) and overall survival (OS). Propensity score matching (PSM) was performed to balance the baseline factors.

**Results:**

According to the multivariate analysis, surgery benefitted both CSS and OS (*P *<* *0.001). After PSM, surgical resection still showed significant benefits in terms of both CSS and OS (*P *<* *0.001). Surgery combined with chemotherapy increased the median CSS by at least 63 months and the median OS by at least 76 months. Subgroup analysis of the Chemo&Surg group revealed that the timing of surgery was not an independent influencing factor for either CSS or OS.

**Conclusions:**

We found that performing additional surgery, in addition to systematic therapy, for GIST liver metastasis resulted in improved CSS and OS. These benefits were not affected by the timing of surgery during systemic treatment.

## Introduction

Gastrointestinal stromal tumors (GISTs) are rare tumors of the digestive system that are believed to originate from mesenchymal Cajal cells. The incidence has been gradually increasing in recent years due to advancements in screening techniques [1, 2]. The metastasis patterns and biological behaviors of GISTs are different from those of other common gastrointestinal malignancies. Previous studies have shown that the liver, rather than the lymph nodes, is the organ most susceptible to GIST metastasis; liver metastasis occurs in approximately 11% of all GIST patients [3] and accounts for 65%–67% of all GIST metastases [4, 5]. Although the Food and Drug Administration approved the first tyrosine kinase inhibitor for treating GIST, imatinib, in 2001, which significantly improved the prognosis of advanced GIST patients receiving systemic treatments [6], most patients with GIST metastasis gradually develop multidrug resistance during long-term systemic treatment [7]. Complete surgical resection of primary GIST with negative margins is recommended in multiple guidelines [8, 9]. However, there is limited clinical evidence to support the efficacy of surgical resection for liver metastases. Apart from systematic treatment, clinical evidence of the effectiveness of surgical treatment for GIST liver metastases is limited.

Given the current lack of evidence regarding the surgical treatment of GIST liver metastases, our objective was to analyze data from the Surveillance, Epidemiology, and End Results (SEER) database using multivariate Cox analysis and propensity score matching (PSM) to evaluate the benefit of surgery during the systematic treatment of GIST liver metastases.

## Methods

### Data sources and inclusion and exclusion criteria

**Figure 1.**
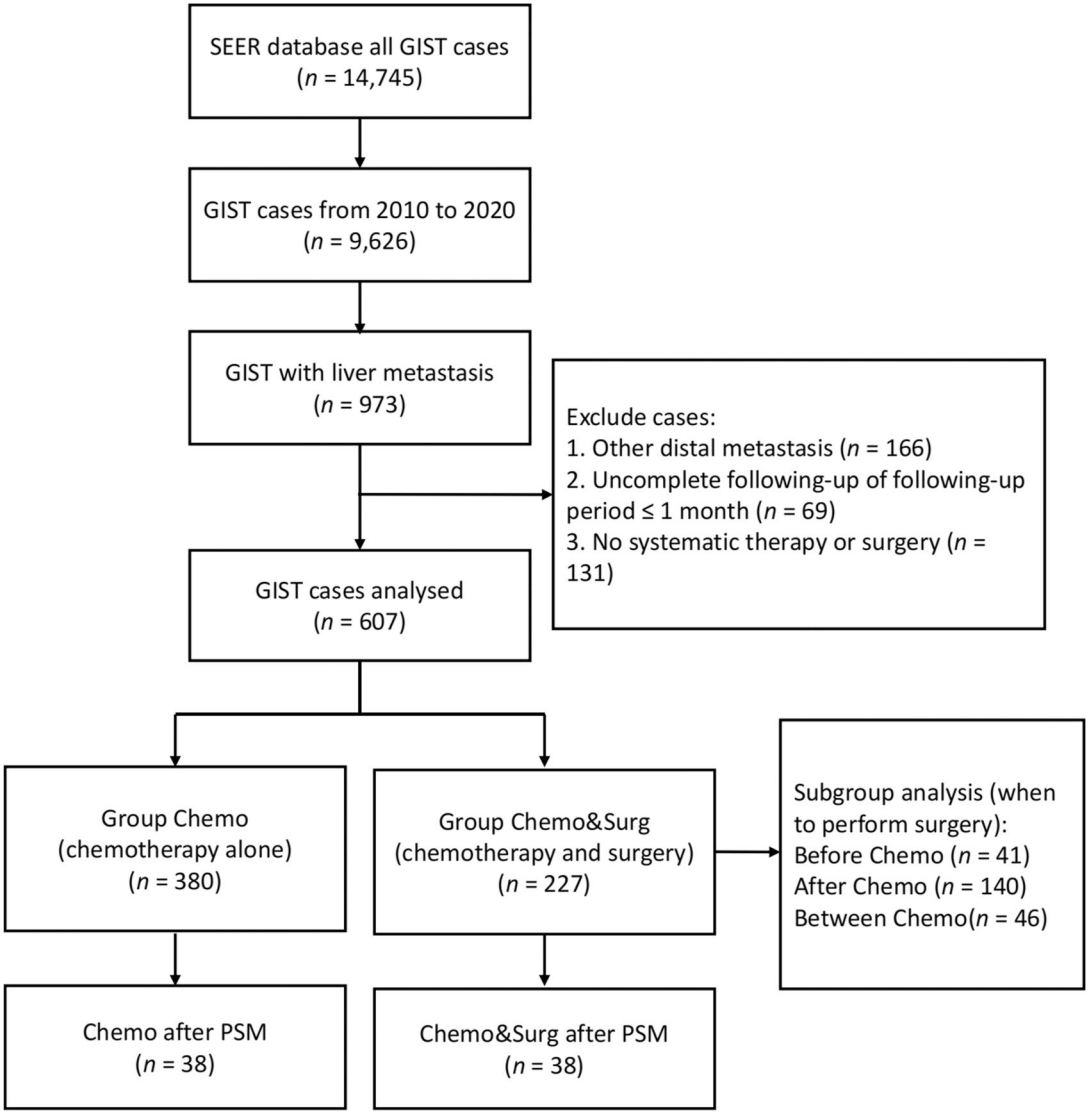
Flow diagram of this study.

The data analyzed in this study were obtained from the SEER database using SEER*Stat 8.4.2. The database was selected from ‘SEER Research Data, 17 Registries, Nov 2022 sub (1990–2020)’. All 14,745 GIST cases were obtained from the ‘AYA site recode 2020 Revision = 4.9 Gastrointestinal stromal tumor, malignant’. Since the SEER database began systematically combining information on liver metastasis in 2010, we subsequently selected 9,626 patients between 2010 and 2020. Among them, 973 patients were identified by screening as patients with liver metastasis according to the ‘SEER Combined Mets at DX-liver’ criteria. The exclusion criteria were as follows: (i) patients with other distant metastases (*n *=* *166); (ii) patients for whom follow-up data were incomplete or follow-up lasted no more than 1 month (*n *=* *69); and (iii) patients for whom no systemic treatment or surgery was performed (*n *=* *131). The remaining 607 patients were ultimately included in this study; 380 patients were treated only with chemotherapy (Chemo group) and 227 patients underwent radical surgery in addition to chemotherapy (Chemo&Surg group), as shown in Figure 1.

### Outcomes and survival analysis

The outcomes of this study were cancer-specific survival (CSS) and overall survival (OS), as recorded in the SEER database. Cox univariate and multivariate regression analyses were used to assess the outcomes. Factors that showed significant differences (*P *<* *0.20) in the univariate Cox analysis were included in the multivariate analysis. The Global Schoenfeld test was used to verify the proportional hazards assumption. This was performed using the ggcoxzph() function in the survminer package of R. The test was conducted to ensure the validity of the Cox analysis in our database (Supplementary Figures S1 and S2).

### Propensity score matching

To further evaluate the effectiveness of surgery on patient survival following basic chemotherapy, we conducted PSM between the Chemo group and the Chemo&Surg group using the Matching and MatchIt packages in the R statistical language. We identified the factors that exhibited a significant difference in descriptive statistics. The standardized propensity score (sPS) in each patient was calculated by using the following formula based on the propensity score (PS):

$$\mathrm{sPS}=0.2\times \sigma (-\log(\frac{1}{PS}-1))$$

The ‘nearest’ matching method and a matching ratio of 1:1 were applied to match the sPSs. The distribution of PSs was used to validate the effectiveness of the matching (Supplementary Figure S3).

### Statistics

Continuous variables are presented as the mean (standard deviation) or median (range). Categorical variables are presented as frequencies and percentages. Baseline characteristics were compared using Student’s *t*-test, the chi-square test, or Fisher’s exact test, as appropriate. Univariate and multivariate analyses were conducted to assess risk factors using Cox proportional hazard models. Hazard ratios (HRs) and 95% confidence intervals (CIs) were calculated. Survival plots were generated by using the Kaplan–Meier method. All the statistical analyses were conducted by using SPSS for Windows (version 26.0; IBM Corp., Armonk, NY, USA) and R for Windows (version 4.3.1; The R Foundation for Statistical Computing, Vienna, Austria). The R packages tidyverse, rms, survminer, and ggplot2 were applied appropriately during the analysis process. A two-sided *P*-value of <0.05 was considered to indicate statistical significance.

## Results

### Analysis of all Chemo and Chemo&Surg cases

Table 1 displays the fundamental clinicopathological characteristics of the two groups. There were no statistically significant differences between the Chemo and Chemo&Surg groups in terms of sex, race, differentiation, primary tumor invasion, or primary tumor size. Patients were older (*P *<* *0.001), mitotic counts were lower (*P *=* *0.040), and primary lesions were more common in the stomach in the Chemo group than in the Chemo&Surg group (*P *<* *0.001). The follow-up period in both groups was sufficient, ranging from 2 to 131 months.

**Table 1.** Basic clinicopathological characteristics of 607 patients with gastrointestinal stromal tumors (GISTs)

Characteristic	Chemo group	Chemo&Surg group	*P*-value	Chemo_PSM group	Chemo&Surg_PSM group	*P*-value
	(*n *=* *380)	(*n *=* *227)		(*n *=* *38)	(*n *=* *38)	
Age (years)^a^			<0.001			0.661
<40	16 (4.2)	22 (9.7)		2 (5.3)	4 (10.5)	
40–59	121 (31.8)	91 (40.1)		12 (31.6)	14 (36.8)	
60–79	190 (50.0)	100 (44.1)		20 (52.6)	18 (47.4)	
>80	53 (13.9)	14 (6.2)		4 (10.5)	2 (5.3)	
Sex			0.452			0.212
Male	226 (59.5)	142 (62.6)		24 (63.2)	29 (76.3)	
Female	154 (40.5)	85 (37.4)		14 (36.8)	9 (23.7)	
Race			0.141			0.547
White	241 (63.4)	160 (71.1)		27 (71.1)	23 (60.5)	
Black	90 (23.7)	40 (17.8)		7 (18.4)	8 (21.1)	
Others	49 (12.9)	25 (11.1)		4 (10.5)	7 (18.4)	
Primary tumor site^a^			<0.001			1.000
Stomach	220 (57.9)	112 (49.3)		34 (89.5)	34 (89.5)	
Small intestine	60 (15.8)	88 (38.8)		4 (10.5)	4 (10.5)	
Colon and rectum	11 (2.9)	11 (4.8)		0 (0.0)	0 (0.0)	
Soft tissue and peritoneum	19 (5.0)	7 (3.1)		0 (0.0)	0 (0.0)	
Others	70 (18.4)	9 (4.0)		0 (0.0)	0 (0.0)	
Differentiation			0.711			0.284
Well differentiated	6 (18.8)	12 (15.2)		2 (14.3)	2 (8.7)	
Moderately differentiated	6 (18.8)	23 (29.1)		2 (14.3)	10 (43.5)	
Poorly differentiated	8 (25.0)	16 (20.3)		4 (28.6)	3 (13.0)	
Undifferentiated	12 (37.5)	28 (35.4)		6 (42.9)	8 (34.8)	
Primary tumor invasion			0.204			–
Localized	36 (56.2)	22 (40.7)		–	–	
Adjacent tissues	6 (9.4)	9 (16.7)		–	–	
Other organs/structures	22 (34.4)	23 (42.6)		–	–	
Primary tumor size (mm)	107.38 ± 67.46	118.66 ± 65.74	0.071	104.86 ± 56.33	116.94 ± 69.06	0.452
Mitotic counts (per 50 HPF)^a^	37.69 ± 41.86	55.06 ± 46.15	0.040	37.37 ± 42.37	37.76 ± 41.40	0.967
Following-up period	28.5 (2–131)	43.0 (2–131)	–	38.0 (6–130)	61.0 (13–130)	–

Chemo = patients with GISTs treated with chemotherapy alone, Chemo&Surg = patients with GISTs treated with chemotherapy and surgery, PSM = propensity score matching, HPF = high-power field.

Continuous variables are expressed as the mean ± standard deviation and were compared by Student’s *t*-test.

Categorical variables are expressed as counts (proportions) and were compared by Pearson’s chi-square test or Fisher’s exact test.

a Factors used for propensity score matching.

We subsequently analyzed CSS and OS in all patients (Table 2). Factors that were considered statistically significant (*P *<* *0.20) in the univariate analysis were included in the multivariate analysis. Specifically, age (*P *=* *0.131), race (*P *<* *0.001), primary tumor site (*P *=* *0.001), and treatment (*P *<* *0.001) were found to be statistically significant in the univariate analysis of CSS and were subsequently included in multivariate analysis. Only treatment (Chemo or Chemo&Surg) was found to be a statistically significant factor (*P *<* *0.001) in the multivariate analysis of CSS. Similarly, age (*P *<* *0.001), race (*P *=* *0.005), primary tumor site (*P *<* *0.001), and treatment (*P *<* *0.001) were found to be statistically significant in the univariate analysis of OS; both age and treatment were found to be statistically significant in the multivariate analysis of OS (*P *<* *0.001). Global Schoenfeld tests on Cox analysis of CSS and OS did not reject the proportional hazards assumption (*P *=* *0.225 and 0.209, Supplementary Figures S1 and S2, respectively).

**Table 2.** Univariate and multivariate Cox analyses of cancer-specific survival and overall survival

Variable	Cancer-specific survival			Overall survival		
	Univariate analysis		Multivariate analysis	Univariate analysis		Multivariate analysis
	HR (95% CI)	*P*-value	*P*-value	HR (95% CI)	*P*-value	*P*-value
Age (years)		0.131	0.368		<0.001	<0.001
<40	Ref.			Ref.		
40–59	1.67 (0.87–3.22)			1.70 (0.91–3.18)		
60–79	1.63 (0.85–3.13)			2.29 (1.24–4.23)		
≥80	2.36 (1.13–4.92)			3.83 (1.98–7.41)		
Sex		0.313			0.757	
Male	Ref.			Ref.		
Female	1.15 (0.88–1.51)			1.04 (0.82–1.31)		
Race		<0.001	0.248		0.005	0.888
White	Ref.			Ref.		
Black	1.89 (1.39–2.55)			1.46 (1.11–1.90)		
Others	0.99 (0.65–1.53)			0.79 (0.54–1.16)		
Primary tumor site		0.001	0.092		<0.001	0.082
Stomach	Ref.			Ref.		
Small intestine	0.69 (0.48–0.99)			0.81 (0.60–1.09)		
Colon and rectum	1.80 (0.84–3.86)			1.44 (1.38–4.31)		
Soft tissue and peritoneum	1.89 (1.10–3.23)			1.61 (0.98–2.66)		
Others	1.45 (1.00–2.10)			1.54 (1.12–2.11)		
Primary tumor size		0.230			0.412	
<50 mm	Ref.			Ref.		
≥50 mm	0.78 (0.51–1.18)			0.86 (0.60–1.23)		
Mitotic counts		0.354			0.332	
<5 counts/50 HPF	Ref.			Ref.		
≥5 counts/50 HPF	1.43 (0.67–3.04)			1.36 (0.73–2.52)		
Treatment		<0.001	<0.001		<0.001	<0.001
Chemotherapy	Ref.			Ref.		
Chemotherapy and surgery	0.47 (0.35–0.63)			0.47 (0.36–0.61)		

HPF = high-power field, HR = hazard ratio, CI = confidence interval.

### Analysis of patients after PSM

**Figure 2.**
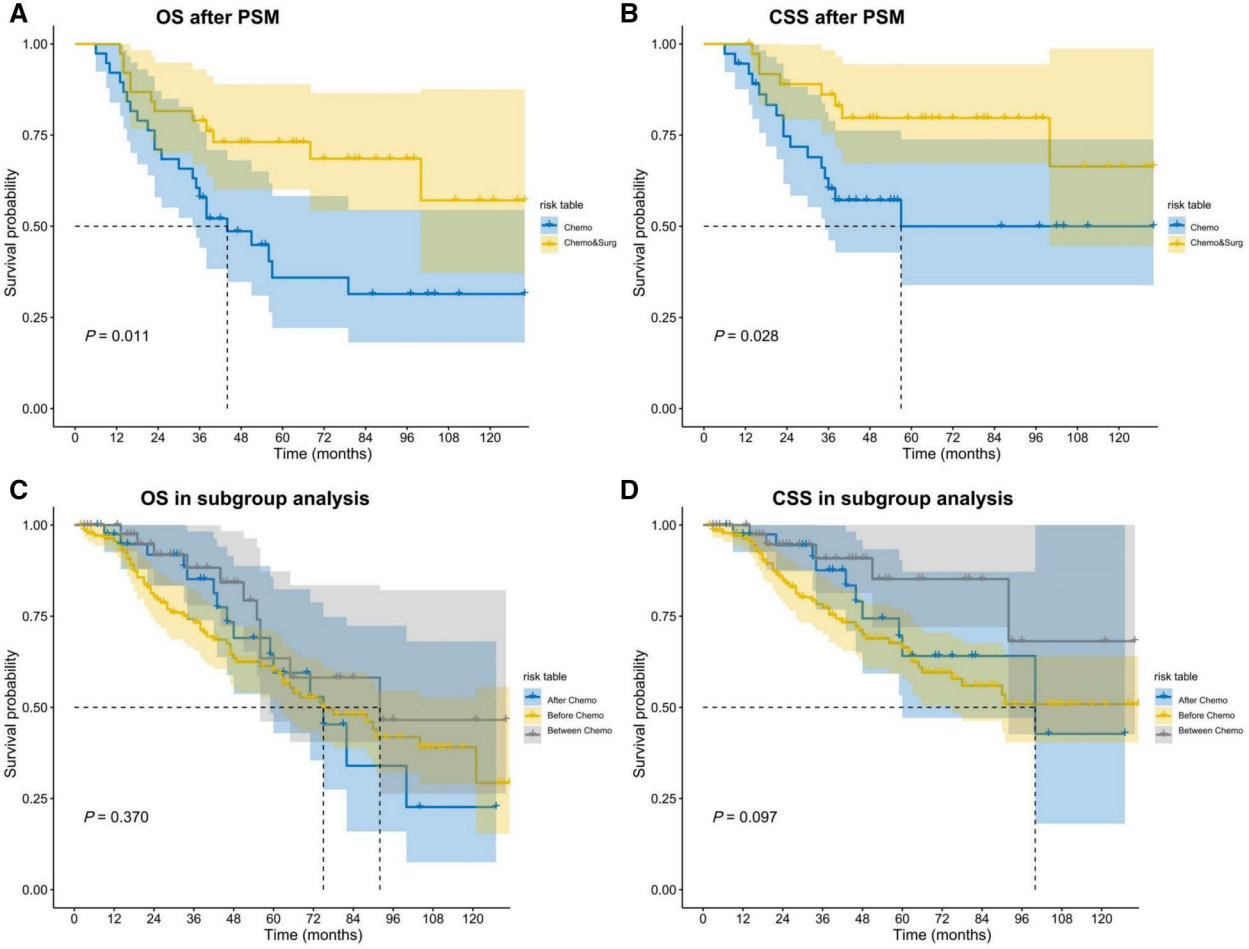
Kaplan–Meier plots of CSS (A) and OS (B) in the Chemo vs. Chemo&Surg groups after PSM. Kaplan–Meier plots of CSS (C) and OS (D) according to subgroup analysis of the timing of surgery during systemic treatment. CSS = cancer-specific survival, OS = overall survival, PSM = propensity score matching.

Age, primary tumor site, and mitotic counts showed different distributions between the two groups (Table 1). These variables were used for PSM to balance the differences in clinicopathological features. After matching the sPSs via PSM, 76 patients remained for further analysis (38 patients in each group; Table 1). We conducted a Kaplan–Meier analysis to compare CSS and OS between the Chemo group with the Chemo&Surg group. The median CSS of the Chemo group was 57 months, with a median OS of 44 months. In contrast, the Chemo&Surg group had a median CSS and OS exceeding 120 months (Figure 2A and B). There were significant differences in both OS and CSS between the two groups (*P *=* *0.011 and 0.028, respectively).

### Subgroup analysis in the Chemo&Surg group

To evaluate the most suitable timing for surgery during the systematic treatment process, we conducted a subgroup analysis within the Chemo&Surg group, which was further divided into three subgroups: after chemotherapy (*n *=* *41), before chemotherapy (*n *=* *140), and between chemotherapy (*n *=* *46). Multivariate Cox analysis was performed to evaluate factors independently associated with survival, including CSS and OS. Race, primary tumor site, and timing of surgery were selected for multivariate analysis of CSS. Age, race, primary tumor site, and timing of surgery were selected for multivariate analysis of OS. The timing of surgery was not found to be an independent influencing factor for either CSS or OS (*P *=* *0.424 and 0.580, respectively; Table 3, Figure 2C and D). Global Schoenfeld tests on Cox analysis of both CSS and OS did not reject the proportional hazards assumption (*P *=* *0.308 and 0.226, Supplementary Figures S4 and S5, respectively).

**Table 3.** Subgroup Cox analysis for the Chemo&Surg group

Variable	Cancer-specific survival			Overall survival		
	Univariate analysis		Multivariate analysis	Univariate analysis		Multivariate analysis
	HR (95% CI)	*P*-value	*P*-value	HR (95% CI)	*P*-value	*P*-value
Age (years)		0.621			0.073	0.082
<40	Ref.			Ref.		
40–59	1.51 (0.58–3.90)			1.43 (0.60–3.41)		
60–79	1.14 (0.43–2.99)			1.54 (0.65–3.64)		
>80	1.89 (0.45–7.94)			3.82 (1.28–11.40)		
Sex		0.408			0.857	
Male	Ref.			Ref.		
Female	1.24 (0.74–2.07)			1.04 (0.67–1.62)		
Race		0.024	0.606		0.030	0.434
White	Ref.			Ref.		
Black	2.18 (1.21–3.92)			1.96 (1.17–3.28)		
Others	0.83 (0.33–2.11)			0.92 (0.44–1.94)		
Primary tumor site		0.053	0.019		0.057	0.052
Stomach	Ref.			Ref.		
Small intestine	1.52 (0.86–2.69)			1.64 (1.03–2.61)		
Colon and rectum	3.83 (1.31–11.14)			3.43 (1.33–8.84)		
Soft tissue and peritoneum	2.80 (0.97–8.11)			1.97 (0.70–5.58)		
Others	2.20 (0.76–6.40)			1.60 (0.57–4.54)		
Primary tumor size		0.501			0.861	
<50 mm	Ref.			Ref.		
≥50 mm	0.76 (0.34–1.69)			0.86 (0.43–1.73)		
Mitotic counts		0.910			0.620	
<5 counts/50 HPF	Ref.			Ref.		
≥5 counts/50 HPF	0.95 (0.39–2.30)			1.22 (0.55–2.72)		
Timing of surgery		0.113	0.424		0.382	0.580
After chemotherapy	Ref.			Ref.		
Before chemotherapy	1.28 (0.65–2.55)			1.09 (0.62–1.92)		
Between chemotherapy	0.49 (0.17–1.43)			0.69 (0.32–1.50)		

HPF = high-power field, HR = hazard ratio, CI = confidence interval.

## Discussion

In this study, we retrospectively collected GIST data from the SEER database, a national database in the USA, spanning the years 2010–2020. This study confirmed that surgical resection of GIST liver metastases during systematic treatment can improve both CSS and OS, regardless of whether surgery is performed before, after, or during systemic treatment.

Our study confirms the role of surgery in the systemic treatment of GIST liver metastases, which aligns with the findings of some previous studies. Du *et al*. [10] compared 41 patients with recurrent or metastatic GIST who underwent surgery during systemic therapy with 210 patients who received only imatinib systemic therapy. The 2-year progression-free survival rate was 31% higher for patients with metastatic GIST who underwent surgery. Shi *et al*. [11] retrospectively analyzed 32 patients with GIST liver metastases who underwent hepatectomy combined with oral tyrosine kinase inhibitor treatment and 98 patients who received only oral tyrosine kinase inhibitors. The study proved that hepatectomy extended the median survival by 36 months [11]. In another retrospective analysis of single-center data from 91 patients with GIST liver metastases, Sutton *et al*. [12] reported that hepatectomy was associated with an improvement in OS, but the benefit of hepatectomy compared with second-line tyrosine kinase inhibitor therapy is still unclear. Previous studies have encountered several disadvantages, including limited sample size, lack of a matching cohort, long duration, and absence of detailed analysis regarding the timing of surgery. Particularly, in previous studies, cases across the past 20 years were collected to ensure a sufficient sample size. However, significant advancements have been made in the targeted drug treatment of GIST in the past decade. As a result, relying on long-term data to assess the effectiveness of surgery for GIST liver metastases may lead to overestimation of its survival advantage. Additionally, the research findings may not be relevant to current patient diagnosis and treatment methods. Therefore, it is essential to use a national database only in recent years to re-evaluate the survival benefits of surgery for patients with GIST liver metastases. Our study matched patients from the Chemo group and the Chemo&Surg group using a large population-based database. The benefits of surgery on CSS and OS were once again confirmed through a matched-group analysis. Furthermore, subgroup analysis demonstrated that these benefits were not affected by the timing of surgery during systemic treatment.

This study has several limitations. First, the SEER database lacks data on several important factors in GIST cases, such as hematological results, degree of enhancement under enhanced computed tomography, and KIT gene expression, all of which have been proven to impact prognosis. Although PSM was used in this study to balance the two groups, bias caused by the aforementioned factors is inevitable. Second, even though this study is a population-based analysis, it is a retrospective study. As a result, the level of evidence in this study is still lower than that of high-quality prospective cohort studies. Even so, this study provides important clinical evidence for the necessity of surgery during the systematic treatment of resectable GIST liver metastases. Particularly, we only used GIST liver metastasis data from the national database for the past 10 years. This makes our conclusions more meaningful in the background of the increasingly effective systemic therapy.

## Conclusions

We conducted a retrospective analysis of a population-based database on GIST liver metastasis and demonstrated that additional surgery, in conjunction with systemic therapy, for GIST liver metastasis improved CSS and OS. These benefits were not affected by the timing of surgery during systemic treatment.

## Supplementary Material

goae095_Supplementary_Data

## Data Availability

All the data analyzed in this study are publicly available from https://seer.cancer.gov/data-software/, as described in the Methods section.
