# Supplementary figures and images for: The benefit of surgery during systematic therapy for gastrointestinal stromal tumor liver metastasis: a SEER-based retrospective study
Source: Gastroenterol Rep (Oxf). 2024 Nov 25;12:goae095. doi: 10.1093/gastro/goae095 (PMC11587995; doi:10.1093/gastro/goae095)

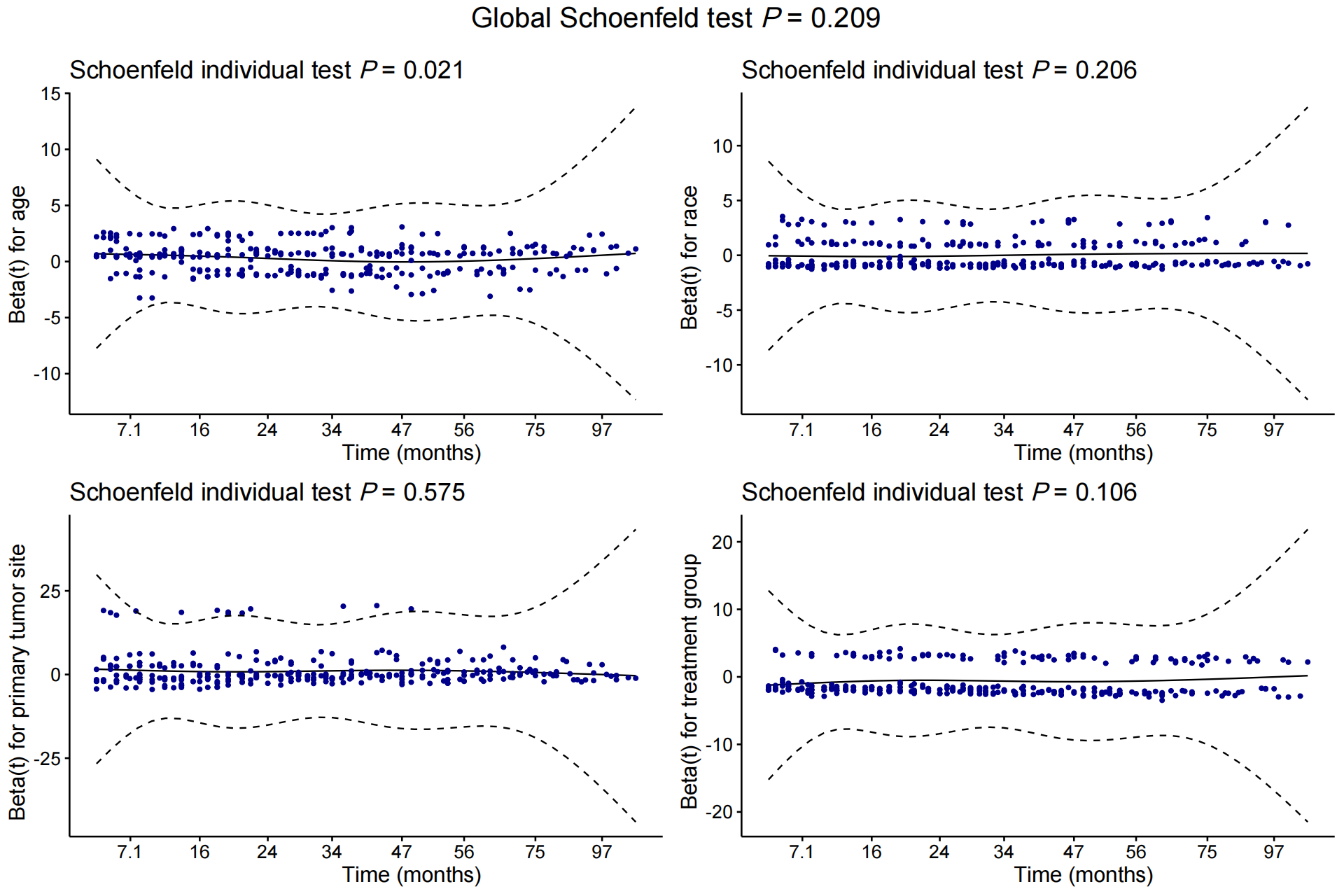

Supplement: goae095_Supplementary_Data [file goae095_supplementary_data.zip › 2024-039 Supplementary Figure S2.tiff]

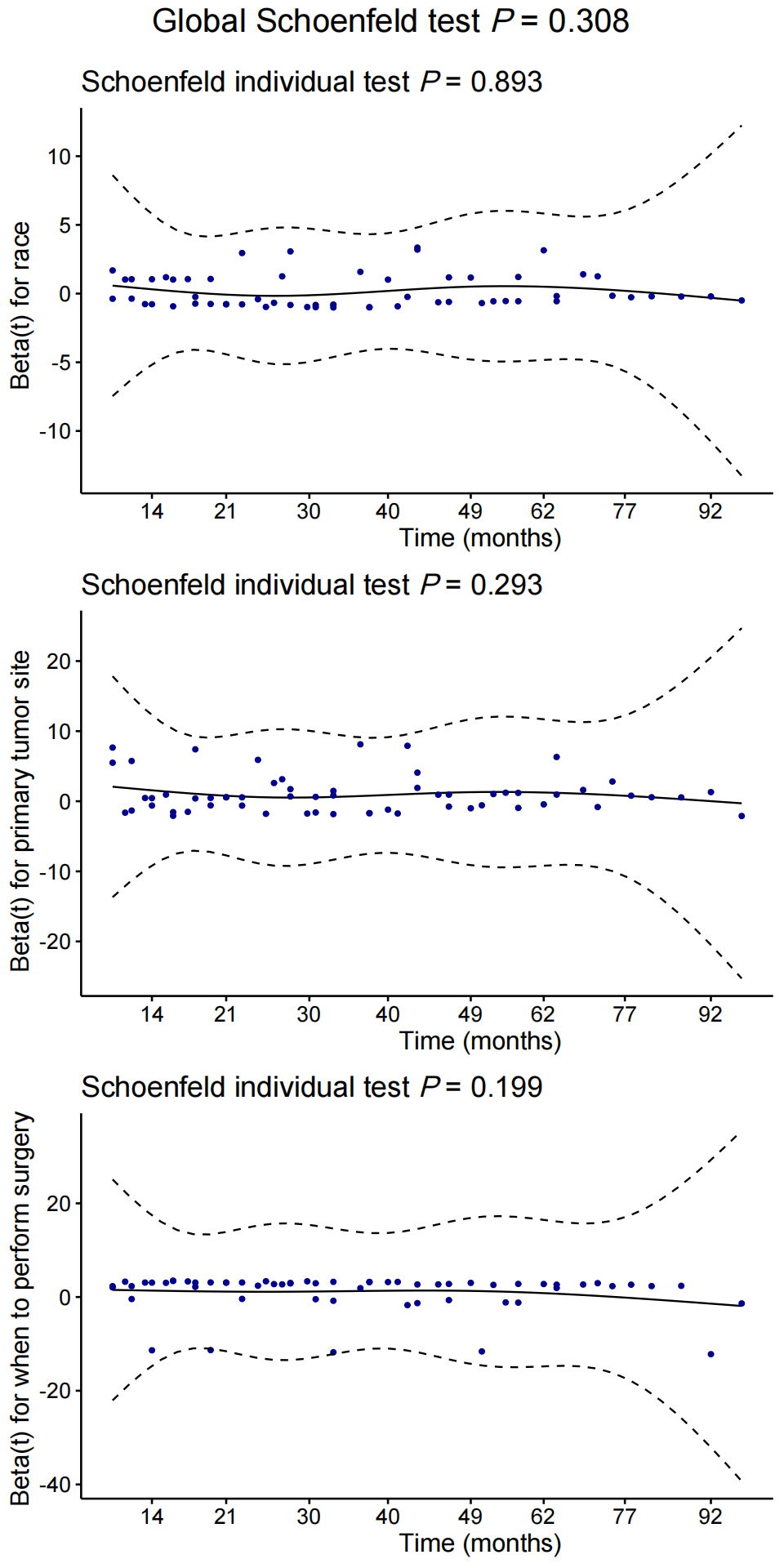

Supplement: goae095_Supplementary_Data [file goae095_supplementary_data.zip › 2024-039 Supplementary Figure S4.tiff]

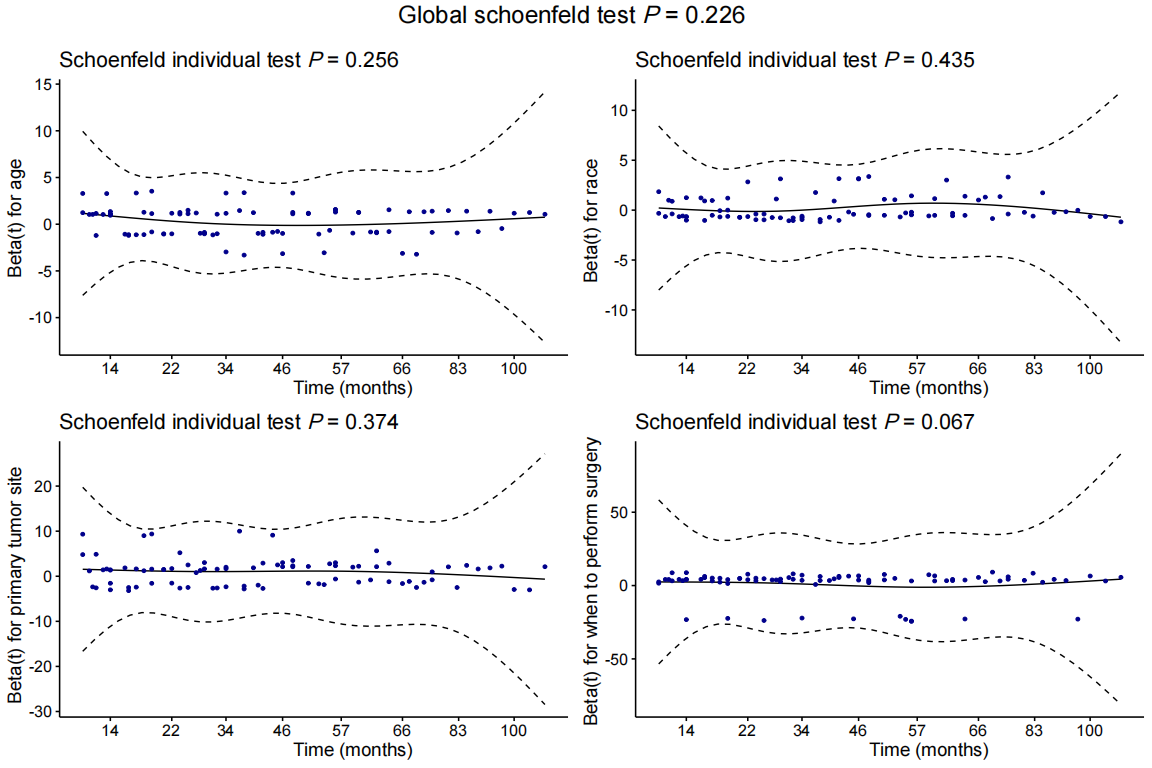

Supplement: goae095_Supplementary_Data [file goae095_supplementary_data.zip › 2024-039 Supplementary Figure S5.tiff]

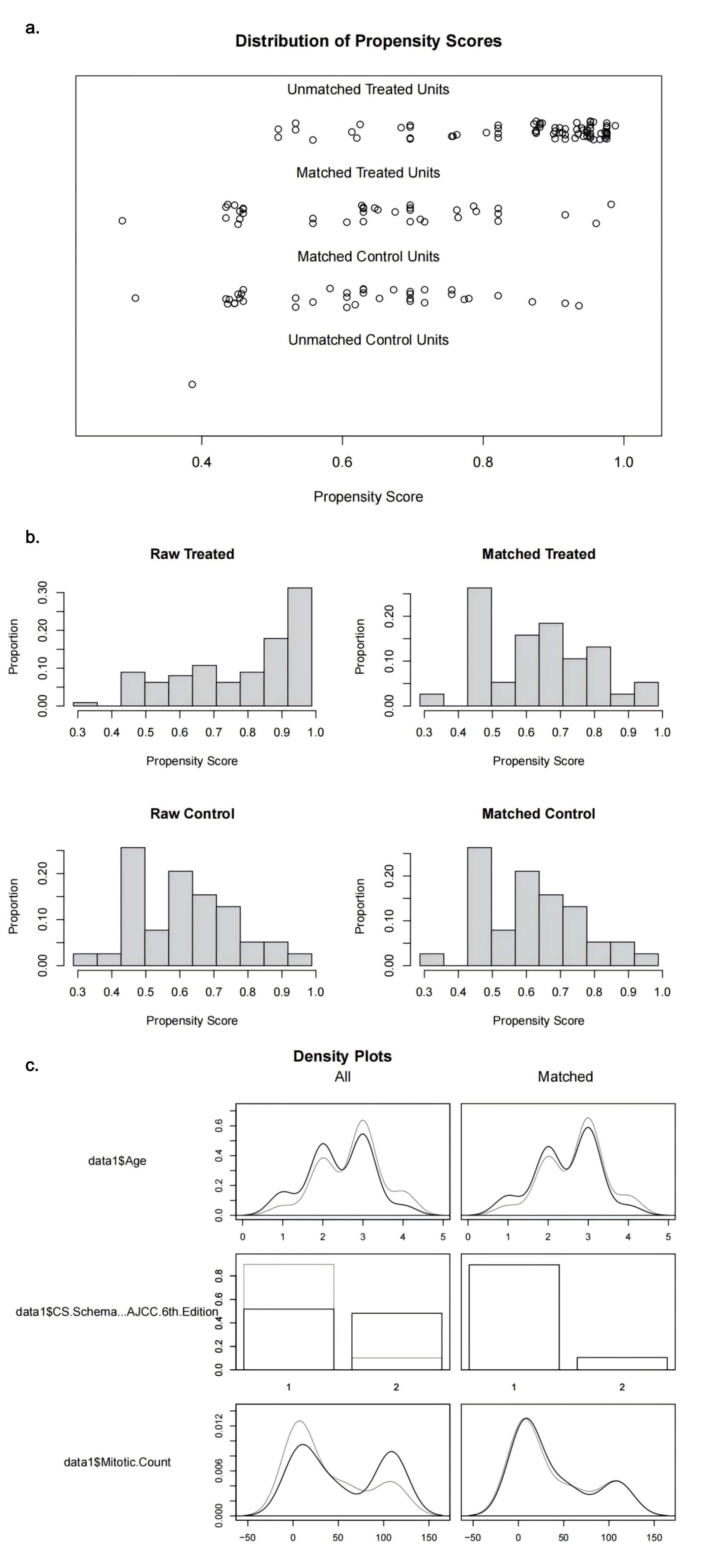

Supplement: goae095_Supplementary_Data [file goae095_supplementary_data.zip › 2024-039 Supplementary Figure S3.tiff]

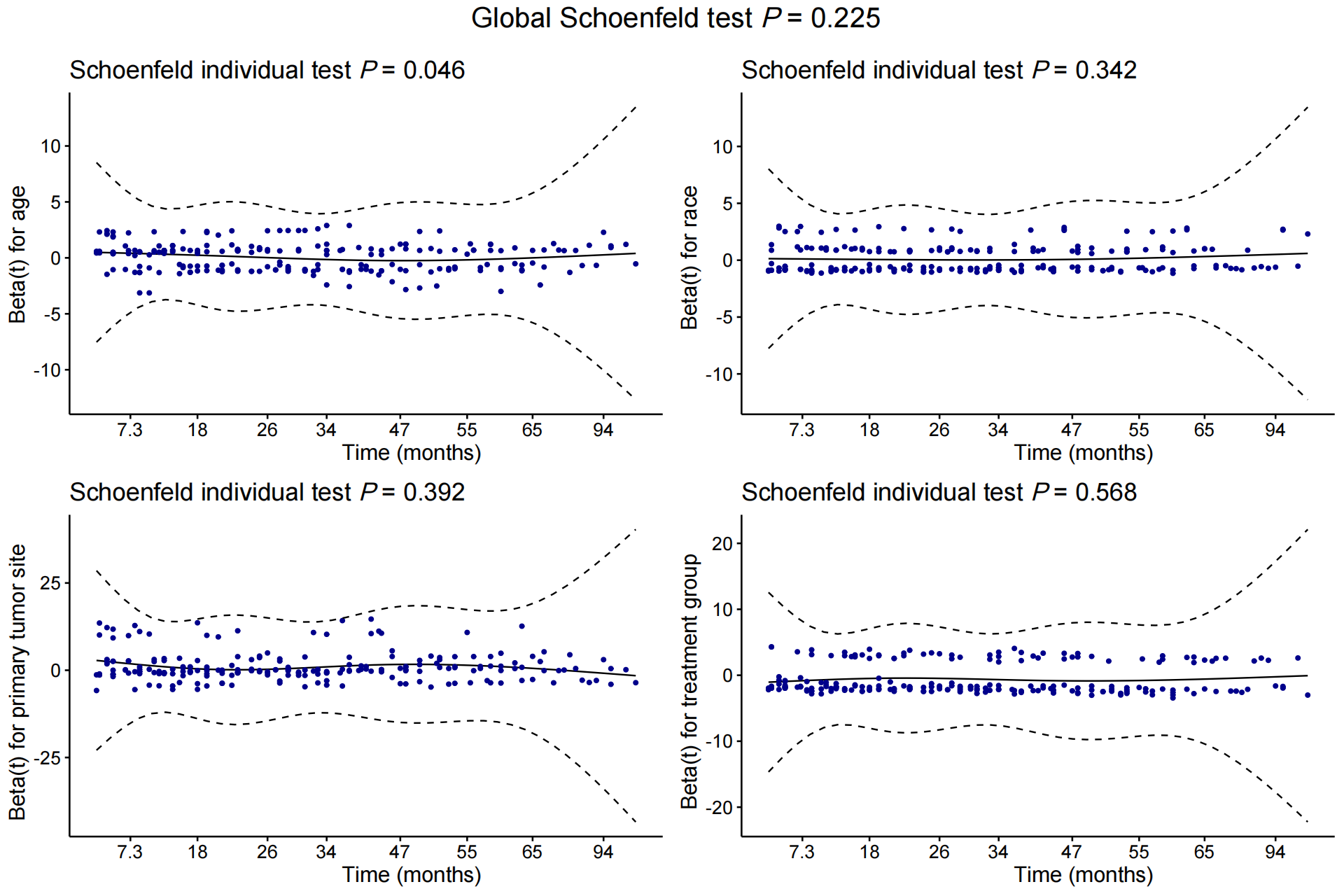

Supplement: goae095_Supplementary_Data [file goae095_supplementary_data.zip › 2024-039 Supplementary Figure S1.tiff]
